# Definitive radiotherapy with stereotactic or IMRT boost with or without radiosensitization strategy for operable breast cancer patients who refuse surgery

**DOI:** 10.1093/jrr/rrac047

**Published:** 2022-07-16

**Authors:** Yuta Shibamoto, Seiya Takano, Masato Iida, Misugi Urano, Kengo Ohta, Masanosuke Oguri, Taro Murai

**Affiliations:** Narita Memorial Proton Center, 78 Shirakawa-cho, Toyohashi, Aichi, 441-8021, Japan; Department of Radiology, Nagoya City University Graduate School of Medical Sciences, 1 Kawasumi, Mizuho-cho, Mizuho-ku, Nagoya, Aichi, 467-8601, Japan; Department of Radiology, Nagoya City University Graduate School of Medical Sciences, 1 Kawasumi, Mizuho-cho, Mizuho-ku, Nagoya, Aichi, 467-8601, Japan; Department of Radiology, Nagoya City University Graduate School of Medical Sciences, 1 Kawasumi, Mizuho-cho, Mizuho-ku, Nagoya, Aichi, 467-8601, Japan; Department of Radiology, Nagoya City University Graduate School of Medical Sciences, 1 Kawasumi, Mizuho-cho, Mizuho-ku, Nagoya, Aichi, 467-8601, Japan; Department of Radiology, Nagoya City University Graduate School of Medical Sciences, 1 Kawasumi, Mizuho-cho, Mizuho-ku, Nagoya, Aichi, 467-8601, Japan; Department of Radiation Oncology, Nagoya Proton Therapy Center, Nagoya City University West Medical Center, 1-1-1 Hirate-cho, Kita-ku, Nagoya, Aichi, 462-8508, Japan; Department of Radiology, Nagoya City University Graduate School of Medical Sciences, 1 Kawasumi, Mizuho-cho, Mizuho-ku, Nagoya, Aichi, 467-8601, Japan

**Keywords:** breast cancer (BC), stereotactic body radiotherapy (SBRT), intensity-modulated radiotherapy (IMRT), tomotherapy, hydrogen peroxide, hyperthermia

## Abstract

For breast cancer (BC) patients who refused surgery, we developed a definitive treatment employing modern sophisticated radiation techniques. Thirty-eight operable BC patients were treated by conventionally fractionated whole-breast (WB) radiotherapy in combination with stereotactic (for primary tumor) or intensity-modulated (for primary tumor with/without regional lymph nodes [LN]) radiotherapy (IMRT) boost. Standard doses were 50 Gy/25 fractions, 21 Gy/3 fractions and 20 Gy/8 fractions, respectively, for the three radiation modalities. Disease stages were 0 (ductal carcinoma *in situ* [DCIS]) in seven patients, I in 12, II in 16 and III in three. In 26 patients, intratumoral hydrogen peroxide injection or hyperthermia with oral tegafur-gimeracil-oteracil potassium (S-1) was also used to sensitize the tumors to radiation. Hormonal and standard systemic therapy were administered in 25 and 13 patients, respectively. Complete and partial responses were obtained in 19 patients each; in patients with partial response, no further regrowth of the residual mass was observed, except for two patients who developed local recurrence. During a follow-up of 8–160 months (median, 50 months for living patients), two, one and two patients developed local relapse, sub-clavicular node metastasis and distant metastasis, respectively. The 5-year rates for overall, progression-free and local relapse-free survival were 97.2, 90.9 and 93.4%, respectively. Fourteen patients developed Grade 3 radiation dermatitis but all recovered after treatment. In 47%, the affected breast became better-rounded, and the nipple of the irradiated breast became higher by ≥1 cm than the contralateral nipple. Our method might be a treatment option for operable BC patients.

## INTRODUCTION

Breast-conserving surgery followed by postoperative radiation therapy (RT) is the standard treatment for early-stage breast cancer (BC). Even with breast-conserving treatment, however, the treated breast deforms and permanent scars remain, sometimes causing pain. Recently, total mastectomy with prosthetic breast reconstruction is also employed for early as well as advanced BC. Despite the development of breast reconstruction, however, the reconstructed breast has an artificial appearance. Therefore, patients’ mental burden and fear of undergoing breast surgery may be substantial and some patients may wish to avoid surgery and conserve their breasts if possible. Regarding non-surgical treatments for operable BC, focused ultrasound and radiofrequency ablation were investigated [1, 2], but such treatments have not been commonly used so far.

RT has been employed for inoperable BC, but the treatments were palliative in most cases [3]. Complete responses were obtained with concurrent chemo-RT at a relatively high rate, but the outcomes appeared worse than those achieved by surgery [4, 5]. Conventional RT was also investigated as a primary treatment for early BC, but the results were incomparable to those obtained by surgery [6]. A trial of carbon-ion RT is ongoing but long-term results are yet unknown [7]. Alternatively, owing to the development of high-precision X-ray RT, early BC might have become curable with a high probability without surgery. With conventional RT alone, administering high enough doses to cure BC might be difficult due to severe skin reactions. However, by employing the modern techniques of stereotactic body RT (SBRT) and intensity-modulated RT (IMRT), cancericidal doses may be delivered with acceptable adverse events.

In 2007, we started a curative treatment for operable BC to comply with the intense desire of patients to conserve their breasts [8]. We elaborated the following treatment protocol: conventional whole-breast (WB) RT at a 50-Gy dose in 25 fractions (fr) is delivered first, and next, boost SBRT is administered in 3 fr (investigated doses: 6–8.5 Gy per fraction) when no lymph node (LN) metastases are detected. When axillary LN metastasis exists or tumor is large and irregular-shaped, IMRT is used for boost irradiation with a dose of 20 Gy in 8 fr, including the involved nodes. Considering the skin toxicity, a higher dose per fraction could not be used for IMRT boost. We previously reported preliminary outcomes in 18 patients [8]; this treatment has been continued with a fixed protocol thereafter. This article reports the updated clinical outcomes in 38 patients undergoing our treatment.

## MATERIALS AND METHODS

### Study design and patient eligibility

The current study was initiated as a dose-seeking pilot study for the SBRT boost. The study details were previously described [8]. After establishing standard doses, the uniform RT protocol has been employed till now, but for larger tumors (usually ≥2 cm in diameter), two radiosensitization strategies (hydrogen peroxide injection prior to irradiation and combination with hyperthermia and oral S-1, tegafur-gimeracil-oteracil potassium) were investigated. An institutional review board approved these studies (approval numbers: NCU-0701, 42-14-0010) and all patients gave informed consent in written form.

Throughout the study, the eligibility criteria included: (i) pathological confirmation of BC, (ii) concrete wish to avoid any surgical procedure, (iii) no hematogenous metastasis on ^18^F-fluoro-deoxyglucose positron emission tomography (FDG-PET), and (iv) no other active malignancy. Pregnant patients and those who had undergone surgery or RT to the breast were ineligible.

### Patients

Between October 2007 and January 2015, nine patients were treated in the study to investigate the optimal SBRT doses, and between July 2015 and June 2019, 15 patients were treated in the pilot study of Kochi Oxydol-Radiation Therapy for Unresectable Carcinomas (KORTUC) treatment employing hydroxy peroxide injection during WBRT [9, 10]. The remaining 14 patients were treated with the established RT protocol with (*n* = 10, between August 2019 and May 2021) or without (*n* = 4, between December 2018 and April 2020) hyperthermia plus S-1. One patient in the initial dose-seeking study had received hyperthermia + S-1, so the patient number in the hyperthermia + S-1 group was 11. All 38 patients were female, with a median age of 49 years (range, 32–83). Characteristics of the patients and their tumors are shown in Table 1.

**Table 1:** Patient and tumor characteristics

Age (years)	Median (Range)	49 (32–83)
Laterality	Right/Left	18/20
Stage^*^	0/IA/IIA/IIB/IIIA/IIIB/IIIC	7/12/9/7/1/1/1
T stage^*^	Tis/T1b/T1c/T2/T3/T4b	7/3/11/14/2/1
N stage^*^	N0/N1/N2/N3	26/11/0/1
TNM classification^*^	TisN0M0/T1bN0M0/T1cN0M0/T1cN1M0/T2N0M0/T2N1M0/T3N1M0/T3N3M0/T4bN1M0	7/3/9/2/7/7/1/1/1
Histology	IDC/DCIS/Scirrhous/Lobular/Unclassified	25/7/3/2/1
Maximum tumor diameter (mm)	Median (range)	21 (6–57)
Hormone receptor	ER (+) and/or PgR (+) /Negative /Unknown	29/6/3
HER2 status	Positive/Negative /Unknown	5/25/8
Subtype	Luminal A/Luminal B/Luminal HER2/HER2/Triple negative/Unknown	11/12/1/4/2/8

IDC, invasive ductal carcinoma; DCIS, ductal carcinoma *in situ*. ER, estrogen receptor; PgR, progesterone receptor. ^*^According to the American Joint Committee on Cancer (AJCC) 8^th^ edition (2018). In the Luminal A group, stages were 0 in two, I in five and II in four. In the Luminal B group, stages were I in four, II in seven, and III in one. The Luminal-HER2 patient had Stage I disease. In the HER2 group, stages were I in one, II in two, and III in one. Two patients with triple negative cancer had Stage 0 and III disease, respectively.

### Treatment

Details of the treatments are summarized in Table 2. First, WBRT was administered using tangential opposed fields up to 50 Gy to isocenter in 25 fr with LINAC 4- or 6-MV X rays. WBRT and LINAC SBRT were planned with Eclipse Ver. 7.5 (Varian Medical Systems, Palo Alto, CA, USA) or RayStation (RaySearch Medical Laboratories AB, Stockholm, Sweden). The superior border of the planning target volume (PTV) was the manubriosternal joint and the inferior border was 1 cm below the inframammary line. The medial border was the midline of the sternum and the lateral border was the midaxillary line, excluding the outermost 2 mm from the skin surface [11]. Regional LN irradiation included the lower part of the ipsilateral axillary LN area (Level I) in all patients. When LN metastases existed, the upper part of the ipsilateral axillary LN area (Level II behind the minor pectoral muscle and Level III when considered necessary) was included. Supraclavicular and internal mammary LNs were not irradiated except for an N3 patient in whom supraclavicular LNs were irradiated. Appropriate angles of two tangential beams were chosen by an attending radiation oncologist. When severe dermatitis developed, reduction of WBRT dose was allowed up to 6 Gy.

**Table 2:** Treatment details

Whole-breast dose (Gy)	50 / 48 / 44	36 / 1 / 1
Radiosensitization method	Hydrogen peroxide injection/Hyperthermia + S-1/None	15 / 11 / 12
Boost method	SBRT / IMRT	22 / 16
Boost modality	LINAC-SBRT/TomoHelical/TomoDirect	17 / 16 / 5
SBRT dose (Gy/fractions)	18/3: 19.5/3: 21/3: 17/2: 25.5/3	2: 1: 15: 2: 2
IMRT dose (Gy/fractions)	17.5/7: 20/8	1: 15
Hormone therapy	Yes: No	25: 13
Chemotherapy^^*^^	Yes: No	3: 35
Anti-HER2 therapy	Yes: No	2: 36

^^*^^Other than S-1.

SBRT was delivered either with a LINAC-based method or tomotherapy; the former procedure employed the published method used for lung tumors [12]. Patients lay in the supine position on an immobilization device. The clinical target volume (CTV) was the visible tumor and LN when present, and the PTV was the CTV with 5–7-mm margins. Registration of the breast contour was performed using computed tomography (CT) in the first and third LINAC-SBRT sessions, and additionally, portal X-ray imaging was performed before each SBRT session. Seven to nine coplanar and non-coplanar beams were used. The dose of LINAC-SBRT was prescribed to isocenter. Respiratory movement of the breast was confirmed to be 5 mm or less in all directions by inspection and fluoroscopy.

Our tomotherapy SBRT or IMRT method was previously described [8, 13]. The fixation method was the same as used in the LINAC-SBRT boost. For IMRT, the PTV was the primary BC and metastatic axillary LN (when present) plus 5–7-mm margins. The dose was prescribed to 50% of the PTV. The TomoHelical mode was employed in 16 patients and the TomoDirect mode in five. The field width was 2.5 and 5 cm in 19 and two patients, respectively; the dynamic-jaw mode was employed in all cases [13]. The pitch was 0.25–0.5 (median, 0.43) and the modulation factor was 1.5–3 (median, 2). Megavoltage CT was performed for registration before each treatment. When severe dermatitis occurred, omission of one SBRT or IMRT session was allowed.

The KOURTC treatment [9, 10] was started at the sixth to eighth fraction of WBRT. Under CT guidance, 6 mL of 0.5% hydrogen peroxide (Oxydol) dissolved in sodium hyaluronate was injected into the center of the breast lesion and whenever possible also into a few other parts around the center twice a week (Monday and Wednesday or Thursday) up to seven or eight times, following the method of Miyatake *et al.* [14] and Ogawa *et al.* [15]. Oxydol injection was not performed during the boost treatment.

The KORTUC treatment became difficult due to the change in medical legislation in 2019. Thereafter, another radiosensitization method (hyperthermia + oral S-1) was employed for tumors ≥ approximately 2 cm. Gimeracil and 5-fluorouracil, a component and metabolic product of S-1, respectively, are reported to have radiosensitizing activity when administered concurrently with radiation [16, 17]. From the evening before the starting day (usually Monday) of irradiation to the morning of weekends (usually Friday), a fluorouracil derivative S-1 was orally administered twice a day, and this was repeated every week until the end of treatment (6–7 weeks in total). The dose was 80 mg per day for body surface area < 1.25 m^2^, 100 mg per day for 1.25 to < 1.5 m^2^, and 120 mg per day for ≥ 1.5 m^2^. Radiofrequency hyperthermia was performed with RF-8 (Yamamoto Vinitor, Osaka, Japan) once weekly, starting on the second week of WBRT. The total session numbers were four or five in 4 or 5 weeks. Thermometry was performed with an apparatus equipped with RF-8, and skin temperature of 40–41.5°C was maintained for at least ≥ 30 minutes.

Neoadjuvant chemotherapy was administered when considered necessary and adjuvant hormonal therapy was administered to hormone-receptor-positive patients, unless the patients refused them; 25 patients received hormonal therapy. Standard adjuvant chemotherapy was recommended, but was performed on a voluntary basis. Systemic neoadjuvant or adjuvant chemotherapy was administered to three patients; used drugs were 5-fluorouracil, paclitaxel, docetaxel, doxorubicine, epirubicine, cyclophosphamide and trastuzumab.

### Follow-up evaluation and statistical analysis

After completion of treatment, the patients visited our hospital at 2- or 3-month intervals until 1 year after RT, 3- or 4-month intervals until 3 years, 4- or 6-month intervals until 5 years and 6–12-month intervals thereafter. At every follow-up visit, physical examination, CT or magnetic resonance imaging (MRI), and a blood test were performed. FDG-PET-CT was performed when necessary. Tumor response to treatment was assessed by Response Evaluation Criteria in Solid Tumors at 6–12 months after RT. Overall survival (OS), progression-free survival (PFS) and local control (LC) rates were calculated by the Kaplan–Meier method from the RT start. We assumed that all of contrast-enhanced CT, contrast-enhanced MRI and FDG-PET-CT were useful to evaluate LC. Common Terminology Criteria for Adverse Events (CTCAE) Ver. 4.0 was used to assess toxicity. The Harvard Scale of Breast Cosmesis [18] was used to evaluate cosmetic outcome at ≥ 9 months after RT. All statistical analyses were carried out using StatView Ver. 5 (SAS Institute Inc., Cary, NC, USA).

## RESULTS

### Patient and treatment characteristics

**Fig. 1:**
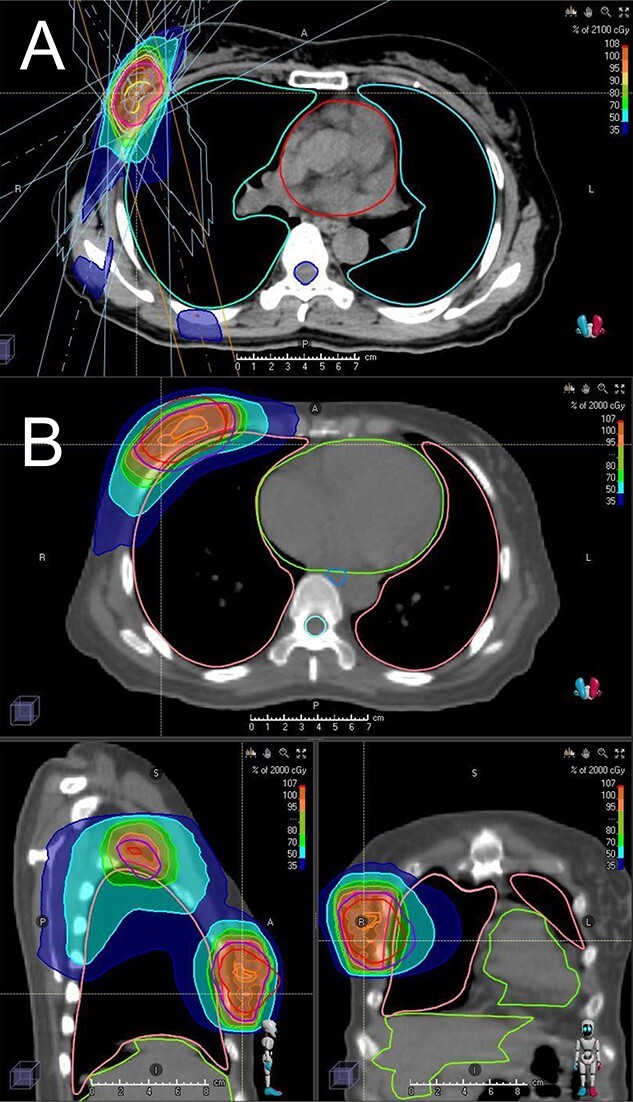
Dose distribution of SBRT boost (A) and IMRT boost (B).

The median tumor size was 21 mm (range, 6–57). Seven patients had a biopsy result of ductal carcinoma *in situ* (DCIS) and they were staged as 0; however, it was unknown whether the biopsy result represented the whole tumor. In two specimens, the main component was DCIS but they contained a part of invasive ductal carcinoma; the tumors were categorized as invasive carcinoma. Actually delivered doses are shown in Table 2. Seventeen patients received LINAC-SBRT boost and 21 received tomotherapy boost. Examples of dose distributions for SBRT and IMRT boost are shown in Fig. 1. Fifteen patients each were treated with 50 Gy plus 21 Gy SBRT boost and 50 Gy + 20 Gy tomotherapy boost. Dose reduction of WBRT (50 to 48 or 44 Gy), SBRT (25.5 Gy/3 fr to 17 Gy/2 fr) and IMRT (20 to 17.5 Gy) was performed in two, two and one patient, respectively. Thirteen patients received standard systemic therapy recommended by the Clinical Practice Guideline of the Japanese Breast Cancer Society (https://jbcs.xsrv.jp/guideline/2018/), whereas 20 patients refused to receive the recommended treatment, especially chemotherapy, although 13 agreed to receive hormonal therapy. In the remaining five patients, standard therapy was unclear due to the lack of information on HER2 and/or hormone receptor status (in three patients with DCIS, standard systemic therapy was considered as hormone therapy although information on HER2 status was lacking). Relationship between stage/subtype and radiation dose/standard systemic therapy is shown in Supplementary Table 1.

### Outcome and toxicity

**Fig. 2:**
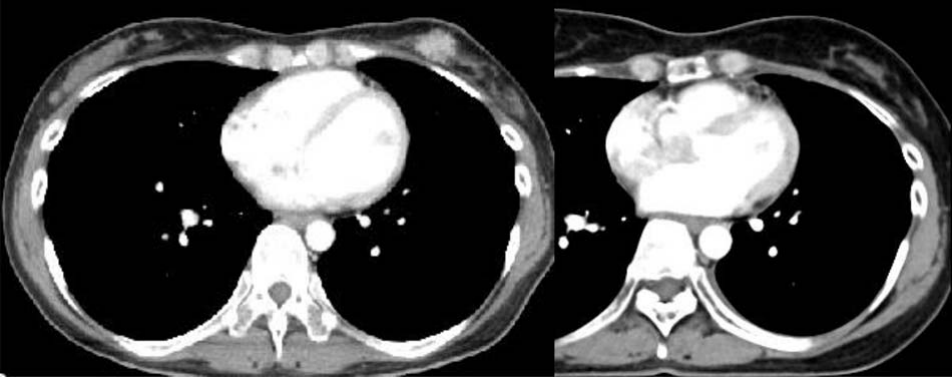
A case showing complete response to treatment (44-year-old woman with invasive ductal carcinoma. Light panel: before treatment; right panel: 5 years after treatment.

The median follow-up period was 50 months (range, 9–170) for living patients. No patients were lost to follow-up. All tumors responded to RT; 19 achieved complete response, while 19 had partial response. An example of complete response is shown in Fig. 2. Two of the latter 19 patients developed local recurrence, but the residual masses in the remaining 17 patients did not show regrowth during follow-up periods of 18–170 months (median, 40 months). Of the two patients, one had a BC measuring 51 mm in maximum diameter before treatment, and the patient underwent our treatment following systemic chemotherapy with 5-fluorouracil, epirubicine and cyclophosphamide. After local recurrence at 25 months, she underwent partial resection of her breast and adjuvant chemotherapy, and was alive without further recurrence at 5 years after the second treatment. Another patient who had had a 21 mm tumor was histologically diagnosed with local recurrence at 12 months after the KORTUC treatment, but she is under observation with hormone therapy without further regrowth of her tumor at 28 months after RT. A residual mass in another patient was biopsied by a surgeon, and the pathological diagnosis was fibroadenoma.

**Fig. 3:**
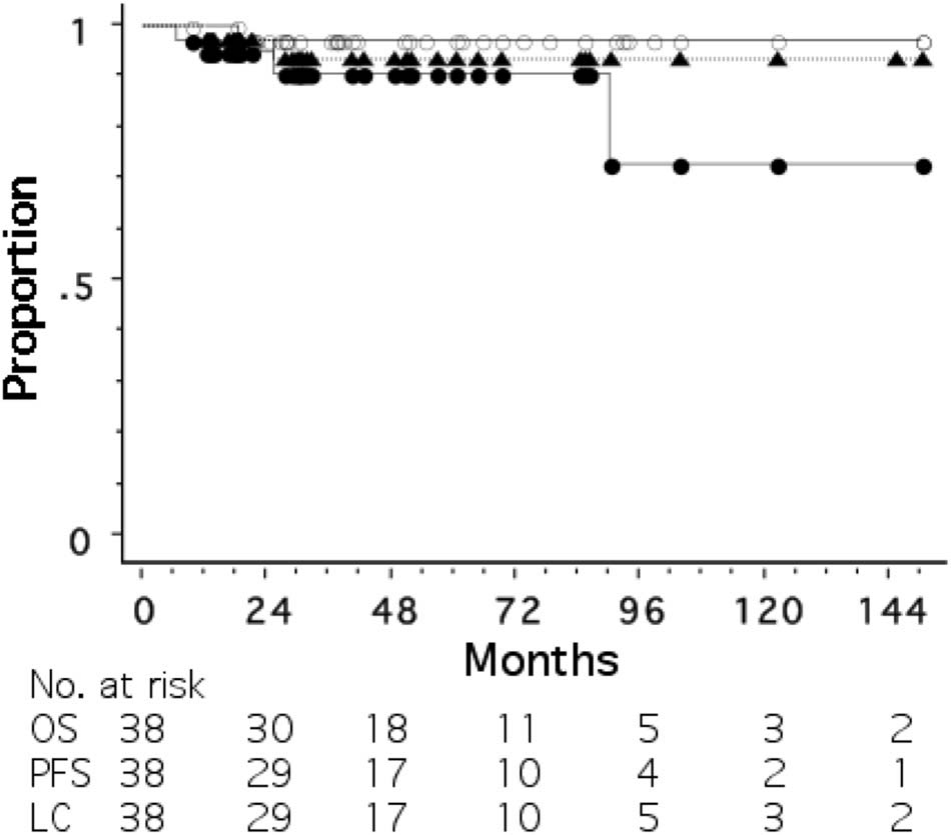
OS (open circle), PFS (open triangle) and LC (closed triangle) curves for all 38 patients.

Patients who developed recurrence are summarized in Table 3. Of the 38 patients, only one patient with stage IIIC (AJCC 8^th^ edition) disease died of pulmonary metastases. Another patient with stage IIB disease developed liver metastasis at 90 months, which was treated by SBRT; she has developed no further recurrence at 61 months after liver SBRT. One patient developed ipsilateral sub-clavicular LN metastasis at 151 months after treatment; she was treated with conventional RT and is in remission at 19 months after second irradiation. Figure 1 shows OS, PFS and LC curves for all 38 patients; the 5-year OS, PFS and LC rates were 97.2, 90.9 and 93.4%, respectively (Fig. 3). Three patients who received standard systemic therapy developed recurrence, while one who did not developed recurrence (Table 3). The 5-year OS, PFS and LC rates were 91, 73 and 80%, respectively, for 13 patients who received standard systemic therapy, and all 100% for 20 patients who did not receive standard systemic therapy (Log-rank *P* = 0.097 for PFS). Treatment outcome in patients with invasive carcinoma who were treated without radiosensitization or with KORTUC is summarized in Supplementary Table 2.

**Table 3:** Patients who developed recurrence

Age	T/N	Size (mm)	Histology	Subtype	Dose (Gy/fr)	Sensitizer	Systemic therapy	Recurrence	Status
41	1c/0	19	Unknown	Unknown	50/25 + 25.5/3	-	-	151 M LN	170 M NED
39	2/1	24	Scirrhous	Luminal B	50/25 + 25.5/3	-	Hormone	90 M Liver	151 M NED
60	3/1	51	IDC	HER2	50/25 + 20/8	-	Chemo + Anti-HER2	25 M Local	83 M NED
									
49	3/3	49	IDC	Triple Neg	50/25 + 20/8	KORTUC	Chemo	6.5 M Lung	18 M Dead
42	2/0	21	Scirrhous	Luminal A	50/25 + 20/8	KORTUC	Hormone	12 M Local	26 M AWD

fr, fractions; M, months; LN, lymph node; NED, no evidence of disease; IDC, invasive ductal carcinoma; Triple Neg, triple negative; KORTUC, Kochi Oxydol-Radiation Therapy for Unresectable Carcinomas; AWD, alive with disease. The third, fourth and fifth patients had received standard systemic therapy, whereas the second patient did not. Standard systemic therapy was unclear in the first patient.

All patients had acute skin toxicity (radiation dermatitis): Grade 1 in 18, Grade 2 in 6 and Grade 3 in 14. All recovered and there was no late skin toxicity, except for Grade 1 telangiectasia, Grade 1 pigmentation and Grade 1 depigmentation seen in two, three and two patients, respectively. In 20 patients with Grade ≥ 2 skin toxicity, 13 and seven had received SBRT and IMRT boost, respectively, and 16 patients had received 50 Gy (WBRT) plus ≥21 Gy/3 fr (SBRT) or 20 Gy/8 fr (IMRT). Sixteen of the 20 patients had received adjuvant systemic therapy. Grade 1 radiation pneumonitis occurred in 22 patients at 2–4 months after completion of RT, which did not seem to be related to systemic therapy. Hydrogen peroxide injection exhibited almost no toxicity, except for small indurations (Grade 1) of the injected sites seen in three patients. Six patients undergoing hyperthermia developed fat induration (Grade 1) in and around their breasts; the indurations almost resolved in 1–2 years in two patients, but remained in four patients. In the other two patients undergoing hyperthermia, burns (Grade 1) were observed, and scars remained thereafter.

### Cosmetic outcome

**Fig. 4:**
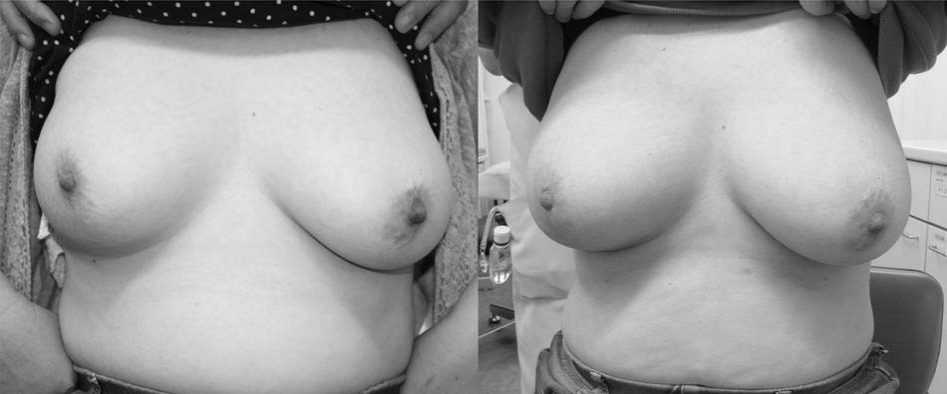
Photographs of a patient at 6 and 12 years after irradiation (left and right panels, respectively). At 6 years, the nipple of the irradiated right breast was located higher than the left nipple, and the two breasts were asymmetric. At 12 years, this asymmetry almost disappeared. The appearance of her breasts did not change greatly when her forearms were lowered.

The Harvard Scale was excellent in 15, good in 22 and fair in 1. We previously reported the enlargement of the irradiated breast, which became better rounded, and the nipple in the affected breast was located at a higher position by ≥ 1 cm than the unirradiated nipple [8]. This phenomenon was observed in 18 of the 38 patients. Such changes in the breast shape became apparent within 1 year after irradiation. Figure 4 shows photographs of a 41-year-old patient (at diagnosis); although there is no picture available before treatment, the irradiated right breast was rounded and the right nipple was located higher at 6 years after treatment (left panel). There was asymmetry between the right and left breasts, but at 12 years after treatment (right panel), this asymmetry became less marked.

## DISCUSSION

Since BC is relatively radiosensitive, it seems reasonable that BCs of a few cm were cured by our high-dose RT. A half of our patients had residual masses on their breasts, and this is in contrast to the finding obtained after carbon-ion therapy; Karasawa *et al.* [7] reported complete response in all patients. This difference might be in part due to how the response was evaluated since it was sometimes difficult to differentiate the residual mass from the breast parenchyma. In our patients with residual masses, they did not show regrowth for a median of 40 months except for two patients with local recurrence, and we think these residual masses were scar-like lesions. Even if local recurrence develops, it might be salvaged by surgery; indeed, one of the patients successfully underwent salvage partial resection. A concern is that, if uncontrolled by our treatment, distant or LN metastasis may develop from residual cancer cells during observation periods after RT. This possibility should be investigated in future. Nevertheless, the high LC and OS rates obtained during this study would indicate that such a possibility is small if any. On the other hand, recent studies suggested that RT might enhance host immunity to tumor cells [19, 20]. If tumors are surgically removed, no gross tumor antigens remain in the body, but after RT, tumor cells may become more antigenic [21] and remain for months until the tumors disappear. As a consequence, small numbers of residual cancer cells in the body might be eradicated by the immune system of the host. If this is true, it can be an advantage of our treatment compared to surgical resection. In the present study, recurrence developed in five patients (three after and one without standard systemic therapy), and 19 patients not undergoing standard systemic therapy have not yet developed recurrence. Therefore, immunological effects of RT should also be investigated in future studies.

The present study confirmed an excellent cosmetic outcome for the irradiated breasts, which we reported previously [8]. In addition to the advantages in terms of the absence of surgical wounds and deformation of the breast due to surgery, the nipple on the treated breast was at a higher position by ≥ 1 cm than the contralateral unirradiated nipple in 47% of our patients. We speculated that this swelling of the treated breast was due to lymph edema induced primarily by WBRT [8]. The finding of lymph edema was not necessarily evident on CT images, but mild edematous changes appeared to be present in many cases. However, asymmetry of the bilateral breasts was a disadvantage in these patients. Interestingly, the swelling of the irradiated breast and asymmetry of the bilateral breasts resolved after 10 years in one patient (Fig. 4). Therefore, her breasts eventually became almost symmetric with no wounds. However, it should be noted that such a phenomenon has been observed in only one patient. In most patients, acute radiation dermatitis was slightly severer than in those undergoing WBRT alone, but all recovered to satisfactory levels. Therefore, our treatment may be an efficient treatment option for women who refuse surgery.

WBRT (50 Gy) plus boost (21 or 20 Gy in 3 or 8 fr) may be sufficient to control small tumors (< 2 cm), but for larger tumors, additional strategies to enhance tumors to the effects of radiation would be necessary to improve LC rates, since the fraction of hypoxic tumor cells increases with tumor enlargement [22, 23]. Various radiosensitization strategies are conceivable, and among them we investigated the KORTUC treatment in 15 patients. In addition to radiosensitization of hypoxic tumor cells, hydrogen peroxide inactivates anti-oxidative enzymes like peroxidases and catalases that are scavengers of radicals produced by radiation and reduce the therapeutic efficacy of RT [24]. Hydrogen peroxide injected twice weekly up to eight times produced no severe adverse events; indurations of the injected sites were the only adverse event. So far, only one patient has developed local recurrence (although the patient is under observation with hormone therapy) and none has developed LN or distant metastases. A Phase I study of this treatment has been completed in the United Kingdom [25], and this treatment will be further investigated in the near future in the world. However, the KORTUC treatment became impossible in 2019 at our institution, so another radiosensitization strategy using hyperthermia and oral S-1 has been evaluated; both have radiosensitizing ability and are expected to potentiate the efficacy of this treatment [16, 17, 26]. The outcome of this alternative strategy to enhance radiation effects is good so far, although the patient number is still small and follow-up period is short. It should also be further evaluated in future studies.

Several limitations exist in this study. In addition to the relatively small patient number and the relatively short follow-up (especially for patients undergoing hyperthermia + S-1 treatment), disease stages were various. The study period was long, and two types of radiosensitization strategies were employed. All of them might influence OS and PFS as well as LC. Therefore, we will continue to accrue more patients to our study and will evaluate outcomes after a longer follow-up period with more patient numbers. Second, our total radiation doses might be relatively high; since successful treatment was reported with lower doses in patients undergoing KORTUC [15, 27], dose reduction may be a topic of future investigation. Finally, we used conventional fractionation for WBRT, but many groups now use hypofractionated WBRT for patients after breast-conserving surgery [28]. So, we will plan to deliver WBRT in 16 fractions in the future studies.

## CONCLUSION

Definitive RT with SBRT or IMRT boost yielded a high LC rate and an excellent cosmetic outcome. In the future, this treatment may become a treatment option in early and middle-stage BC patients who wish to avoid surgery. Further clinical evaluation is warranted.

## Supplementary Material

Suppl_Table_1_Rev_rrac047

Suppl_Table_2_Rev_rrac047
