# Supplementary material for: Definitive radiotherapy with stereotactic or IMRT boost with or without radiosensitization strategy for operable breast cancer patients who refuse surgery
Source: J Radiat Res. 2022 Jul 16;63(6):849–55. doi: 10.1093/jrr/rrac047 (PMC9726698; doi:10.1093/jrr/rrac047)
Supplement: Suppl_Table_1_Rev_rrac047 [file suppl_table_1_rev_rrac047.docx]

**Supplementary Table 1.** Relationship between stage/subtype and radiation dose/standard systemic therapy

Stage *n* Radiation dose (Gy/fraction) *n* Standard systemic therapy

(Whole breast and SBRT or IMRT)

1. 5 50/25 and > 21/3 or 20/8 5 +

2 <50/25 and/or <21/3 or <20/8 1 -

1 Standard therapy unclear

I 9 50/25 and >21/3 or 20/8 2 +

3 <50/25 and/or <21/3 or <20/8 9 -

1 Standard therapy unclear

II 15 50/25 and >21/3 or 20/8 4 +

1 <50/25 and/or <21/3 or <20/8 9 -

3 Standard therapy unlcear

III 3 50/25 and >21/3 or 20/8 2 +

1 -

Sybtype *n* Radiation dose (Gy/fraction) *n* Standard systemic therapy

(Whole breast and SBRT or IMRT)

Luminal A 9 50/25 and >21/3 or 20/8 7 +

2 <50/25 and/or <21/3 or <20/8 4 -

Luminal B 11 50/25 and >21/3 or 20/8 0 +

1 <50/25 and/or <21/3 or <20/8 12 -

Luminal – HER2 1 50/25 and >21/3 or 20/8 1 -

HER2 3 50/25 and >21/3 or 20/8 2 +

1 <50/25 and/or < 21/3 or <20/8 2 -

Triple negative 1 50/25 and >21/3 or 20/8 1 +

1 <50/25 and/or < 21/3 or <20/8 1 -

Unknown 7 50/25 and >21/3 or 20/8 3* +

1 <50/25 and/or <21/3 or <20/8 5 Standard therapy unclear

*In the 3 patients with DCIS and unknown HER2 status, hormonal therapy was performed.
