# Supplementary material for: Definitive radiotherapy with stereotactic or IMRT boost with or without radiosensitization strategy for operable breast cancer patients who refuse surgery
Source: J Radiat Res. 2022 Jul 16;63(6):849–55. doi: 10.1093/jrr/rrac047 (PMC9726698; doi:10.1093/jrr/rrac047)
Supplement: Suppl_Table_2_Rev_rrac047 [file suppl_table_2_rev_rrac047.docx]

**Supplementary Table 2.** Summary of outcome in patients with invasive carcinoma who were treated without radiosensitization or with KORTUC

Sensitizer *n* Stage Subtype Standard systemic 5-year OS 5-year PFS 5-year LC

(I/II/III) (A/B/LH/H/TN/UK) therapy (+/-/UC) (%) (%) (%)

None 11 7/3/1 4/4/0/2/0/1 2/8/1 100 90 90

KORTUC 13 2/10/1 3/4/1/1/1/3 5/5/3 92 85 92

KORTUC, Kochi Oxydol-Radiation Therapy for Unresectable Carcinomas; OS, overall survival; PFS, progression-free survival; LC, local control; A/B/LH/H/TN/UK, Luminal A/Luminal B/Luminal HER2 /HER2/Triple negative/Unknown; UC, unclear.
